# St. Dunstan's Methods for Civilian Institutions

**Published:** 1919-07-19

**Authors:** 


### THE NEW TREATMENT OF THE BLIND.

### St. Dunstan's Methods for Civilian Institutions.

In his fourth report on the work of St. Dunstan's Hostel for Blinded Soldiers and Sailors, Sir Arthur Pearson leaves aside the interesting story of the way in which other senses are cultivated to take the place of the sense of sight. The previous reports have been full of information on this matter, and the whole is collected in his new book, "Victory over Blindness." The latest report contents itself with a survey of the sports of the patients and with the guest-house and centre of training, to which in all 1,483 patients have come. Of these nearly half have completed their re-education, and are now at work in the world on the occupations which they have mastered. The remaining moiety is engaged in the St. Dunstan's workshops. There is, it is true, a section, numbering less than 4 per cent., which has proved incapable of taking a self-supporting life in the world. The problem ahead is that of the man with one eye or partly damaged sight, who is expected, sooner or later, to stand in need of the training provided at St. Dunstan's. The men of the Navy had this compensation for their risks in war, that loss of sight was not among them. The sailors at St. Dunstan's are the men of the R.N.V.R., who were fighting in the trenches.

It is interesting to note the classes and occupations from which the blinded patients were chiefly drawn, and to compare with them the occupations which experience shows to offer the best chance of success. Miners, farm labourers, mill hands, and regular soldiers were the four classes of men most in evidence. The openings for which blind men have the best chances of success are eight: Massage; office work and typing; telephone-operating; the crafts of the cobbler, the basket-maker, and the mat-maker; joinery and poultry-farming. For additional work in spare moments, netting, which is elaborately taught, is the most profitable. For those who have lost a limb as well as their sight, occupation has been found in the ranks of tobacconists and newsagents. With the pension, described by Sir Arthur as "good," the men who do not prefer to take things easily can generally, we are told, earn more than they were able to do before they lost their sight. Even when compared with the earnings in the same occupation of men with the full use of their eyes, the incomes of St. Dunstan's patients are good. Because the training of the blind is more arduous, the concentration more intense, the result of the training is apt to be a skill which many normal men cannot rival. As for the one-third and one-half of the patients who were originally or have become married, it has been found that "the help and encouragement" of a wife can hardly be exaggerated.

But the test of the success here claimed is provided by the letters which Sir Arthur has received from former patients who are now "on their own." They have been started; the moment has come when the value of the training and its commercial return has been put to the proof. The success of the blind masseur is one with which hospital men are familiar. Indeed blind masseurs are sometimes preferred. Just as they sometimes head the lists of examinees, and do better than the man with the full use of his eyes, so they become and retain the chief posts as heads of departments. In private practice, once doctors can recommend them from personal experience, the blind masseur is also successful. Any prejudice against him quickly vanishes when the patient finds him almost as independent as himself. From this it is but a step to regard the blindness of a masseur as a recommendation. The subtlety of their sense of touch has to be experienced to be believed. It is a discovery. Sir Robert Jones, who employs them at his orthopædic institution at Alder Hey, Liverpool, believes, too, that they have "an extraordinarily good psychological effect upon their patients."

The basket-makers, "without exception," have never wanted work, and the fact that purchases now have to be carried home has created a demand for shopping-baskets all over the country. The general market seems to be steady, and repeat orders are the rule. It is noteworthy that the men take a craftsman's pride in their work, and have a keen sense of "the standard of St. Dunstan's," which they feel to be as high as any in the trade.

The mat-makers suffer from a rise in the cost of materials, but since mat-making is generally combined with boot-repairing, a fluctuating trade, the two occupations generally suffice to give a proper return to the worker. The men who are to settle in the North are now being taught clogging, and the extent of the demands for boot-repairing can be judged from the man who says that in the first twenty weeks of his occupation he finished one thousand pairs of boots. The joiners and picture-framers, who add to this the sale of artist's colours, report a steady flow of orders, and the poultry-farmers in particular excite admiration from the way in which they find their way about the farms, while those in business in London soon find their way across the City without difficulty. The chief fact which the Hostel's inspectors report is the tendency of the men to work too long, and since all work done by the blind is largely brain work they have to be reminded of the need for open-air exercise and recreation.

In the professions, to which the officers especially are drawn, we read of a teacher who has passed the intermediate examination in Arts of London University, and is now reading for his final. He was allowed no longer time for his papers than any other candidate, and the officers include a barrister, a clergyman in charge of a Midland parish, and a third who is in charge of the after-care department at St. Dunstan's, on the importance of which we have often remarked. Many officers are poultry-farmers. One whose stock of birds and apple-trees has increased from 20 to 300, expects with ordinary

**The New Treatment of the Blind—(*continued*).**

luck to make a sum equal to his pension. His staff consists of two girls, both being trained by himself, and occasionally an odd man for heavy digging. The farm now includes lavender and mushrooms. The most remarkable instance is that of a young scientific chemist, who was injured while perfecting a new form of high explosive, by an explosion which killed everyone else in the laboratory and blinded him. He has, after a course at St. Dunstan's, returned to the firm for whom he was working when the accident occurred, and is now resuming his technical work.

The future entirely depends on the survival of the after-care system. That is the bulwark on which the success of the blinded men reposes. They are, after training, normal men with a difference. The difference is latent, but it is there. To prevent their relapse, they must have the help and encouragement of the inspectors, who can encourage the markets as well as the men, and secure continuity of employment by supplementing the local market by the sale of unsold goods at central depôts. We have gone into these details because the work of St. Dunstan's will not have finished when the last military patient has come and gone. Its work and success are already reacting on institutions for civilians who are blind. The new treatment invented at St. Dunstan's is of general application, and its authors have done for the blind a work comparable to that achieved a century ago by Pinel for mental patients.

